# Cortisol administration after extinction in a fear-conditioning paradigm with traumatic film clips prevents return of fear

**DOI:** 10.1038/s41398-019-0455-0

**Published:** 2019-04-08

**Authors:** Alexandra H. Brueckner, Johanna Lass-Hennemann, Frank H. Wilhelm, Diana S. Ferreira de Sá, Tanja Michael

**Affiliations:** 1 Division of Clinical Psychology and Psychotherapy, Department of Psychology, Saarland University, Saarbrücken, Germany; 2 Division of Clinical Psychology, Psychotherapy and Health Psychology, Department of Psychology, University of Salzburg, Salzburg, Austria

## Abstract

Cortisol is a stress hormone and potent modulator of learning and memory processes. If administered after learning, cortisol can enhance memory consolidation. Yet it is unknown whether cortisol administration after fear extinction learning strengthens extinction memory. Extinction is a crucial mechanism underlying psychotherapy of posttraumatic stress disorder (PTSD). The present study examined whether extinction can be enhanced by administering cortisol after extinction training. In a registered, randomized, double-blind and placebo controlled trial, 50 healthy participants were exposed to a differential fear-conditioning paradigm with neutral faces as conditioned stimuli (CS) and traumatic film clips as unconditioned stimuli (US). They received either cortisol (*n* = 25) or placebo (*n* = 25) immediately after extinction. The cortisol group showed less fear during a return of fear manipulation (reinstatement) evidenced by attenuated fear potentiated startle responses and US-expectancy ratings than the placebo group. Results indicate that cortisol administration after fear extinction strengthens extinction memory and suggest that it might be advantageous to administer cortisol subsequent to successful exposure treatment sessions.

## Introduction

Exposure-based therapies are effective treatment approaches for posttraumatic stress disorder (PTSD)^1^. However, many patients still suffer from PTSD after treatment^2^ and treatment is associated with high dropout rates^3^. Fear extinction is thought to be one of the mechanisms underlying the effectiveness of exposure therapies^4,5^. During fear extinction a previous fear-laden stimulus is presented without aversive consequences. Thus, during extinction learning a new extinction memory trace is formed^6^ that is no longer associated with fear. However, the old fear-laden memory trace remains intact and extinguished fear responses can return^7,8^. Thus, recent research has focussed on possible enhancers of extinction learning as they may boost the effectiveness of psychotherapy for PTSD.

The glucocorticoid cortisol has been proposed as one possible enhancer of extinction learning^9^. Cortisol is well-known for its memory modulating effects; it enhances the consolidation of newly acquired memories and inhibits the retrieval of previously learned material^10^. Thus, cortisol may act on exposure therapy (1) by promoting the consolidation of extinction learning, but also (2) by inhibiting fear memory retrieval. Indeed, animal studies have shown that glucocorticoids play an important role in successful fear extinction^11–14^. However, only few studies examined the effect of cortisol on fear extinction in humans^15–17^. Two studies investigated the effects of heightened cortisol levels prior to extinction learning. Bentz and colleagues^16^ showed that endogenously heightened cortisol levels prior to extinction training led to reduced conditioned fear in a memory retrieval test in men. However, a study by Merz and colleagues^15^ administering cortisol prior to extinction training found impaired fear extinction in men. It is important to note that both studies could not disentangle the effects of cortisol on fear retrieval, extinction memory acquisition or extinction memory consolidation. Hamacher and colleagues^17^ investigated the effect of a stress procedure on extinction memory consolidation. They found a context-dependent stronger return of fear in the stress group compared with the control group. However, the authors did not directly assess cortisol effects on extinction memory consolidation, but looked at more general stress effects on extinction memory consolidation.

To summarize, it remains unknown whether cortisol influences extinction by promoting the consolidation of extinction learning and/or by inhibiting fear retrieval. Relevantly, several clinical studies have shown that exogenous cortisol administration, as well as high endogenous cortisol levels enhance the success of exposure treatment in patients with different anxiety disorders^18–23^ and PTSD^24,25^. However, these studies also cannot distinguish between the two cortisol effects (inhibited fear retrieval and/or better consolidation of new no-fear memory acquired in exposure), as cortisol levels were enhanced before and during exposure. In summary, although cortisol seems a promising psychopharmacological adjunct to exposure therapy, it needs to be established whether it acts by suppressing fear memory retrieval, by enhancing consolidation of extinction learning, or by a combination of both processes.

Thus, in a registered, randomized, double-blind, and placebo controlled trial, we tested the hypothesis that cortisol enhances the consolidation of fear extinction memory. Fifty participants underwent a differential fear-conditioning paradigm with neutral faces as conditioned stimuli (CS) and traumatic film clips as unconditioned stimuli (US). We chose these film clips as US since they have higher comparability with real traumatic events than classical US like electric shocks. Further, recent studies demonstrated that such films are powerful US in conditioning studies^26–29^. The experiment consisted of three conditioning phases applied on different days: acquisition (day 1), extinction (day 2), and a return of fear (ROF) manipulation with reinstatement followed by a ROF test (day 3). Importantly, cortisol/placebo was administered solely subsequent to extinction training, i.e., we directly tested the hypothesis that cortisol enhances the consolidation of extinction learning. Primary outcome measure was the fear response during ROF test. Fear was assessed both on a physiological (fear potentiated startle, FPS; skin conductance response, SCR) and a subjective level (US expectancy and valence ratings). We expected the cortisol group to exhibit lower fear responses during ROF test than the placebo group.

## Material and methods

### Participants and general procedure

Seventy-three healthy, non-smoking students (44 females) with a body mass index (BMI) within the normal range (women: 18.5–26 kg/m^2^, men: 19–27 kg/m^2^) participated in the study. Sample size was based on previous studies examining cortisol effects^15,30,31^. In order to minimize the influence of menstrual cycle phase on hormonal status, only women with regular use of monophasic androgenic oral contraceptives were included. Contraceptives containing drosperinone (e.g., Yasmin, Yasminelle, or Petibelle) were also an exclusion criteria due to their effect on the endogenous cortisol synthesis^32^. Exclusion criteria were a history of systemic or oral cortisol therapy, any current medication and/or drug intake, current mental and/or physical illness, previous physical and/or sexual abuse, known pregnancy and lactation, and participation in a pharmacological study within the past month. Participants were instructed to refrain from physical exercise, alcohol, and smoking during the experimental days, as well as from caffeine beverages three hours prior to the experimental sessions. All participants provided written informed consent and received 50 Euros as reimbursement. The study was registered in the German Clinical Trial Register (DRKS00010684) and approved by the local ethics committee.

In a double-blind design participants were randomly assigned to the cortisol or the placebo group, filled out several questionnaires prior to and at the end of testing and assessed the emotional impact of the study participation at the end of the study and two weeks later (to be reported elsewhere). Focus of the current study is the conditioning procedure.

### Conditioning procedure

**Fig. 1:**
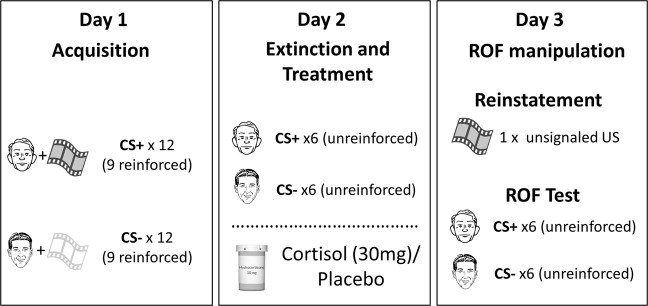
Experimental design. The experiment included three appointments on three consecutive days: acquisition (day 1), extinction (day 2), and return of fear (ROF) manipulation and ROF test (day 3). Cortisol (30 mg) or placebo was administered directly after extinction training

The differential fear-conditioning task took place on three consecutive days: Acquisition training to establish conditioned fear on day 1, extinction training and subsequent cortisol/placebo intake on day 2, and reinstatement and ROF testing on day 3 (see Fig. 1). Each conditioning phase started with nine startle-probe habituation trials, pre-ratings of US-expectancy and valence of the CSs, followed by a randomized order of trials of each CS-type (reinforced CS+ presented with a traumatic film clip (US), CS- presented with a neutral film clip as control condition (CC), and unreinforced CS+/CS− never paired with US/CC). During each trial the startle probe was presented 7 s after CS onset and inter-trial intervals (ITIs) varied between 15 and 20 s. Physiological measures were continuously recorded throughout each phase. US-Expectancy and valence for the CSs were rated at the beginning, halfway through and at the end of each phase. To control for diurnal variations in cortisol levels, all experimental sessions were scheduled between 1 p.m. and 6 p.m.

#### Day 1: Acquisition

**Fig. 2:**
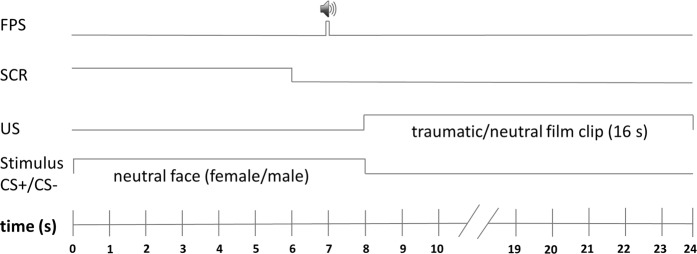
Reinforced conditioning trials. CS duration was 8 s. Startle probe was presented 7 s after CS onset. At CS offset either a traumatic film clip (US) or a neutral film clip as control condition (CC) was presented for 16 s

After participants were prepared for physiological measures (FPS, SCR), they were reminded that they could discontinue participation at any time and requested to put on headphones. Further, they were informed that in the following session two faces would be presented repeatedly and that one of the faces could be followed by an aversive film clip whereas the other face could be followed by a neutral film clip. They were asked to continuously watch attentively the events on the screen without closing their eyes and to memorize what they have learned. During acquisition training, CS+/CS− were each presented 12 times for 8 s, reinforcement rate was 75%. Immediately after CS presentation, the US/CC followed (see Fig. 2) and in case of an unreinforced CS+/CS− trial the participant saw a gray screen for 16 s.

#### Day 2: Extinction and cortisol/placebo administration

To ensure memory consolidation of the acquired fear association, the extinction procedure took place 24 h after acquisition. Participants were told that they would be presented with the same stimuli as on the previous day. CS+/CS− were each presented 6 times for 8 s and were never followed by the US/CC. This rather short extinction phase was chosen to avoid floor effects for the extinction training, as we were interested in the enhancing effects of cortisol on the consolidation of extinction learning. Immediately following extinction participants received either cortisol or placebo and provided saliva samples prior to (pre-treat) and 30 min after pill intake (post-treat).

#### Day 3: Reinstatement and return of fear (ROF) test

Twenty-four hours after cortisol/placebo administration the presentation of one US (reinstatement) was realized followed by the ROF test. For each participant the US was the last traumatic film clip that was presented in the acquisition phase. CS+/CS− were each presented 6 times for 8 s and never followed by US. Participants provided 5 saliva samples: 1 upon arrival (arrival-rei), 1 prior to reinstatement (pre-rei), and 3 after reinstatement test in order to assess the stress reaction in response to the reinstatement procedure (post-rei, +15 min, +30 min). At the end of the experiment, participants received financial reimbursement and were encouraged to contact the experimenter in case they felt any kind of uneasiness or distress related to the experiment.

### Stimuli

#### Conditioned stimuli

The CS were four different frontal view images of female or male Caucasian faces (Radboud Faces Database) with neutral facial expressions matched for picture quality (525 × 675 pixel) and valence (no.23: M = 51.67, no.33: M = 48.30, no.61: M1 = 50.35, no.31: M2 = 50.76). To select these faces, in a pilot study 46 participants rated 40 neutral faces (20 female) regarding their valence using a visual analogue scale (VAS) ranging from 0 (not at all unpleasant) to 100 (very unpleasant). In the conditioning procedure each participant was either presented two male or two female faces as CSs, which was counterbalanced between groups (cortisol vs. placebo) and sex (women vs. men). Further, the stimuli were presented in a pseudo-randomized fashion with a balanced number of trial-type (CS+/CS− and reinforced/unreinforced) over the first and second half of the experiment and with the restriction that no more than two trials of the same type should appear in a row.

#### Unconditioned stimuli/control condition

Nine traumatic 16-s film clips (with their original sound) displaying sexual or physical violence were used as US to simulate the confrontation with anxiety-inducing content as naturally as possible. The US was presented immediately at CS+ offset (see Fig. 2). As a control condition (CC) the CS− was followed by neutral film clips (matched to the traumatic film clips for the number of people interacting with each other and film quality). All film clips were generated from different commercial feature films (supplementary Information) and some have been employed in previous studies^26,33^.

### Cortisol/placebo administration

Participants received 30 mg cortisol (3 pills hydrocortisone 10 mg; Galen, Kiel, Germany) or placebos (3 pills P-Tabletten Lichtenstein; Winthrop, Frankfurt am Main, Germany) immediately after extinction learning. The dose of cortisol was based on previous studies examining cortisol effects in fear-conditioning paradigms^15,30,31^.

### Behavioral outcome measures

US-expectancy ratings were assessed with the question “How much do you expect the next presentation of this face to be followed by an aversive film clip?” using a VAS ranging from “very low expectancy” to “very high expectancy” (0–100). For valence ratings participants were asked to rate “How unpleasant is this face for you?” using a VAS ranging from “not at all unpleasant” to “very unpleasant” (0–100), while the CS was presented on the screen. For analysis, ratings prior to each phase (pre-acq, pre-ext, pre-ROF) were compared to the ratings in the middle (peri-acq, peri-ext, peri-ROF) and at the end of each conditioning phase (post-acq, post-ext, and post-ROF).

### Physiological outcome measures

Physiological data were recorded by ActiveTwo Software (BioSemi, Amsterdam, Netherlands) and further analyzed with Autonomic Nervous System Laboratory (ANSLAB) version 2.6^34^. For outlier analysis, SCR and FPS were z-standardized. Outliers were defined for each participant separately over all data (*Z* > 3). Outliers and missing data due to technical difficulties were replaced by linear trend at point for experimental phase (acquisition, extinction, reinstatement) and stimulus type separately^35–37^. For analysis, we averaged the physiological data of the first half of the trials (early) and of the second half of the trials (late) in each conditioning phase.

#### Fear potentiated startle (FPS)

Startle response was measured from orbicularis oculi electromyogram and amplitude values were calculated relative to the baseline of the signal 50 ms before the trigger onset. FPS responses were normalized by T-transformation. Four participants showed less than 70% valid trials and were excluded from further analysis regarding FPS.

#### Skin conductance response (SCR)

SCR was calculated by subtracting the average pre-CS baseline skin conductance level (SCL) (−2 to 0 s relative to CS onset) from the maximum CS SCL (0 to 6 s relative to CS onset, to minimize startle-probe artefacts). SCR data was normalized by using the natural logarithm of 1 + SCR (in µS).

#### Saliva samples

Saliva samples were collected using Salivette® tubes (Saarstedt, Nümbrecht, Germany) and kept at −20 °C until analysis at the cortisol laboratory of the University of Trier (details on the biochemical analysis, see^38^). Intraassay variability was between 4% and 6.7% and interassay variability between 7.1 and 9%, respectively.

### Statistical analysis

Data were analyzed with SPSS (IBM SPSS Statistics 21). The alpha level was set at *p* < 0.05. Assumptions for statistical analysis (e.g., normal distribution, estimate of variance) have been verified and, if necessary, corrections or alternative procedures were applied. Greenhouse-Geisser corrected *p*-values are reported if the assumption of sphericity was violated, effect sizes are reported as partial *η*^2^ and post hoc analysis are performed with *t*-tests.

## Results

### Participant characteristics

Reporting discomfort from watching the traumatic film clips, 11 participants dropped out during or after the fear acquisition phase. Twelve additional participants did not acquire CS-US contingency and were excluded from further analysis. Note that—for the excluded participants—there was also no evidence for implicit awareness of CS-US contingency neither for FPS (no main effect of CS-Type: *F*_*1,10* _= 1.4, *p* = .264, non-significant CS-Type*Time: *F*_*11,110* _= 1.11, *p* = .361) nor for SCR (no main effect of CS-Type: *F*_*1,13* _= 0.37, *p* = .555, non-significant CS-Type*Time: *F*_*11,143* _= 0.69, *p* = .622).^35^ The final sample consisted of 50 participants, 25 per group (for participants’ characteristics, see Table 1).

**Table 1:** Participants‘ characteristics in the cortisol and the placebo group

	Cortisol group *(n* = *25)* *M(SD)*	Placebo group *(n* = *25)* *M(SD)*	*p*-value
Sex (female/male)	14/11	11/14	0.774
Age	24.60 (4.33)	23.88 (3.00)	0.498
BMI	22.11 (2.25)	22.48 (2.46)	0.576
BDI	3.71 (4.86)	4.64 (5.16)	0.519
STAI-T	32.88 (8.52)	34.68 (10.89)	0.522
Cortisol concentration (post-extinction/pre-treatment)	3.71 (4.86)	3.52 (2.02)	0.313

### Startle habituation

The habituation of the startle response at the beginning of each conditioning phase was tested with a mixed design ANOVA with the factors Trial (1st, 2nd, …, 9th) and Group (cortisol, placebo). Participants in both groups habituated to the startle probe prior to acquisition (Trial: *F*_8,368_ = 13.81, *p* < .001, *η*^2^ = .23), extinction (Trial: *F*_8,344_ = 8.82, *p* < .001, *η*^2^ = .17), and reinstatement (Trial: *F*_8,352_ = 12.12, *p* < .001, *η*^2^ = .22) in absence of any group-related effects (all *p*s > .154).

### Manipulation checks

#### Cortisol treatment

A mixed design ANOVA with the factors Time (pre-treat, post-treat) and Group (cortisol, placebo) revealed elevated cortisol levels after cortisol intake in the cortisol group as compared with the placebo group (Time*Group: *F*_1,46_ = 30.27, *p*. < 001, *η*^2^ = .40).

### Acquisition

#### Behavioral outcome measures

We conducted mixed design ANOVAs with the factors CS-Type (CS +, CS-), Time (pre-acq, peri-acq and post-acq), and Group (cortisol, placebo). Analysis for US-expectancy revealed effects for CS-Type (*F*_1,46_ = 230.22, *p* < .001, *η*^2^ = .83), Time (*F*_2,92_ = 9.55, *p* < .001, *η*^2^ = .17), Time*CS-Type (*F*_2,92_ = 179.12, *p* < .001, *η*^2^ = .80) and Time*CS-Type*Group (*F*_2,92_ = 5.58, *p* = .007, *η*^2^ = .11), but no further interaction effects involving the Group factor (all *p*s > .192). Post hoc analysis revealed a significant difference regarding CS+ vs. CS− at end of acquisition phase (*p* < .001), indicating successful learning. The interaction with the group factor was due to a difference between the groups regarding the CS+ at pre-acquisition (*p* = .034; i.e., lower CS + ratings in the cortisol group), which was no longer present at the end of acquisition (*p* = .383). (Descriptive data of the CS+ in the acquisition phase: cortisol group pre-acq = 32.93 (29.19), peri-acq = 81.07 (29.17), post-acq = 84.51 (23.40); placebo group pre-acq = 50.53 (27.80), peri-acq = 79.48 (26.17), post-acq = 89.32 (14.08)). Analysis of valence ratings revealed effects for CS-Type (*F*_1,48_ = 38.19, *p* < .001, *η*^2^ = .44), Time (*F*_2,96_ = 9.12, *p* < .001, *η*^2^ = .16), CS-Type*Time (*F*_2,96_ = 40.01, *p* < .001, *η*^2^ = .46), CS-Type*Group (*F*_1,48_ = 5.24, *p* = .026, *η*^2^ = .10) and CS-Type*Time*Group (*F*_2,96_ = 6.51, *p* = .008, *η*^2^ = .12). Successful learning was indicated by the significant post hoc test comparing CS+ and CS− at the end of the acquisition phase (*p* < .001). The interaction with Group was due to a baseline difference between placebo and cortisol groups regarding the CS− prior to acquisition (*p* < .001; i.e., lower CS− ratings in the placebo group), which was no longer present at the end of acquisition (*p* = .093). (Descriptive data of the CS− in the acquisition phase: cortisol group pre-acq = 40.81 (23.23), peri-acq = 24.38 (21.96), post-acq = 23.19(24.20); placebo group pre-acq = 15.43 (18.6), peri-acq = 19.24 (20.53), post-acq = 16.09 (17.25)).

#### Physiological outcome measures

Mixed design ANOVAs with the factors Group (cortisol, placebo), CS-Type (CS+, CS−), and Time (early (trials 1–6), late (trials 7–12)) were conducted. Analysis for FPS revealed effects for CS-Type (*F*_1,46_ = 10.01, *p* = .003, *η*^2^ = .18) and Time (*F*_1, 46_ = 33.62, *p* < .001, *η*^2^ = .42), and no effects for CS-Type*Time (*F*_1,46_ = 2.97, *p* = .092) and for Group related effects (all *p*s > .085). Post hoc analysis revealed stronger FPS responding to the CS+ than to the CS− at late acquisition (*p* < .001), indicating successful learning. SCR analysis displayed an effect for CS Type (*F*_1,48_ = 11.42, *p* < .001, *η*^2^ = .19), and no effects for Time (*F*_1,48_ = 0.96, *p* = .331), CS-Type*Time (*F*_1,48_ = 0.79, *p* = .379) and no effects for Group (all *ps* > .064). Post hoc analysis revealed larger SCR responses to the CS+ than to the CS− at late acquisition (*p* = .008), indicating successful learning.

### Extinction

#### Behavioral outcome measures

We conducted mixed design ANOVAs with the factors CS-Type (CS+, CS−), Time (pre-ext, peri-ext, and post-ext), and Group (cortisol, placebo). Analysis for US-expectancy showed effects for CS-Type (*F*_1,47_ = 150.04, *p* < .001, *η*^2^ = .8), Time (*F*_*2*,94_ = 7.65, *p* < .001, *η*^2^ = .14), Time*CS-Type (*F*_2,94_ = 18.52, *p* < .001, *η*^2^ = .28), Time*Group (*F*_2,47_ = 5.66, *p* = .005, *η*^2^ = .11), Time*CS-Type*Group (*F*_2,94_ = 3.46, *p* < .001, *η*^2^ = .07), and no interaction effect for CS-Type*Group (*F*_1,47_ = 714.81, *p* = .470). Post hoc analysis revealed significantly decrease in both groups for the CS+ from the beginning to the end of extinction (*p* < .001), but at post extinction there is still a differential effect between CS+ and CS− (*p* < .001), indicating incomplete extinction learning. To follow up on the effects for Group, post-hoc analysis between the CS+ trials at post extinction revealed a stronger extinction response in the cortisol group than in the placebo group (*p* = .010).

The valence ratings analysis revealed an effect for CS-Type (*F*_1,47_ = 64.4, *p* < .001, *η*^2^ = .58), and no effects for time (*F*_*2*,94_ = 0.4, *p* = .675), CS-Type*Time (*F*_2,94_ = 0.79, *p* = .456) and no effects for Group (all *p*s < .113), showing no extinction learning regarding valence.

#### Physiological outcome measures

We conducted mixed design ANOVAs with Group (cortisol, placebo) as between subject factor, and CS-Type (CS+, CS−) and Time (early (trials 1–3), late (trials 4–6)) as within-subjects factors. Analysis for FPS revealed an effect for Time (*F*_1,42_ = 48.35, *p* < .001, *η*^2^ = .54), no effects for CS-Type (*F*_1,42_ = 2.44, *p* = .126), CS-Type*Time (*F*_1,42_ = 1.64, *p* = .21), CS-Type*Group (*F*_1,42_ = 1.36, *p* = .250), Time*Group (*F*_1,42_ = 0.91, *p* = .346), and a marginal significant effect for CS-Type*Time*Group (*F*_1,42_ = 3.816, *p* = .057). The marginally significant three way interaction was due to a stronger extinction response in the placebo group than in the cortisol group. Post hoc analysis revealed no differential FPS responding at late extinction (*p* = .222), indicating successful extinction learning.

SCR analysis found no effects for CS-Type (*F*_1,44_ = 1.37, *p* = .247), for Time (*F*_1,44_ = 2.81, *p* = .101), for Time*CS-Type (*F*_1,44_ = 0.21, *p* = .648) and no interaction involving the Group factor (all *p*s > .129). Even though there was no longer a differential effect regarding CS+/CS−, the missing Time effect does not allow to conclude that extinction learning was successful.

### Tests of hypothesis–return of fear test

#### Behavioral outcome measures

**Fig. 3:**
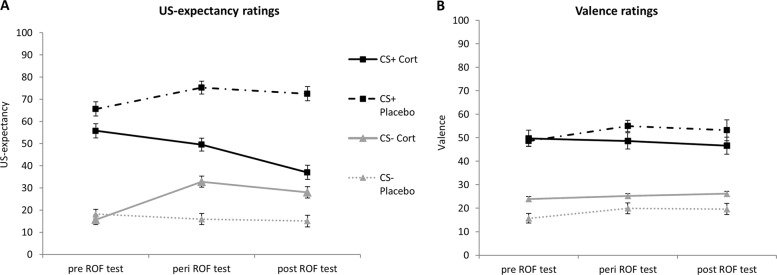
Behavioral outcome measures. **a** US-Expectancy ratings for CS+ and CS− during ROF test for cortisol and placebo group **b** valence ratings for CS+ and CS− during ROF test for cortisol and placebo group (means +/− standard errors)

To test our hypothesis that cortisol administration leads to lower ROF, we conducted mixed design ANOVAs with the factors CS-Type (CS+, CS−), Time (pre-ROF, peri-ROF, and post-ROF) and Group (cortisol, placebo). Regarding US-expectancy, effects for CS-Type (*F*_1,44_ = 67.52, *p* < .001, *η*^2^ = .61), CS-Type*Group (*F*_1,44_ = 12.32, *p* < .001, *η*^2^ = .22), and Time*CS-Type*Group (*F*_2,88_ = 14.10, *p* < .001, *η*^2^ = .24) were significant. To ensure that our cortisol effect for the expectancy ratings is not explained by the stronger extinction response for US-expectancy ratings in the cortisol group, we repeated the analysis with the expectancy for the CS+ at the end of extinction as a covariate. The relevant Time*CS-Type*Group (*F*_2,86_ = 15.71, *p* < .001, *η*^2^ = .27) interaction remained significant (see Fig. 3a). Post hoc analysis confirmed our hypothesis by revealing that US-expectancy for the CS+ decreased from pre-ROF to post-ROF in the cortisol group (*p* = .002), whereas in the placebo group US-expectancy for the CS+ remained high (*p* = .083). In addition, the cortisol group showed no differential response to the CS+/CS− at post-ROF (*p* = .197) in contrast to the placebo group (*p* < .001). Explorative analysis with sex as additional factor did not reveal any sex-related cortisol effects regarding expectancy ratings (Time*Group*Sex: *F*_2,84_ = 0.08, *p* *=* .885, CS-Type*Group*Sex: *F*_1,42_ = 0.66, *p* *=* .420, Time*CS-Type*Group*Sex: *F*_2,84 _= 1.09, *p* *=* .328). Analyses for valence ratings revealed effects for Time (*F*_2,88_ = 4.33, *p* = .016, *η*^2^ = .09) and CS-Type (*F*_1,44_ = 45.53, *p* *<* .001, *η*^2^ = .51), but the relevant Time*CS-Type*Group interaction (*F*_2,88_ = 1.99, *p* = .143) was not significant (see Fig. 3b). Thus, our hypothesis was not confirmed with respect to valence ratings.

#### Physiological outcome measures

**Fig. 4:**
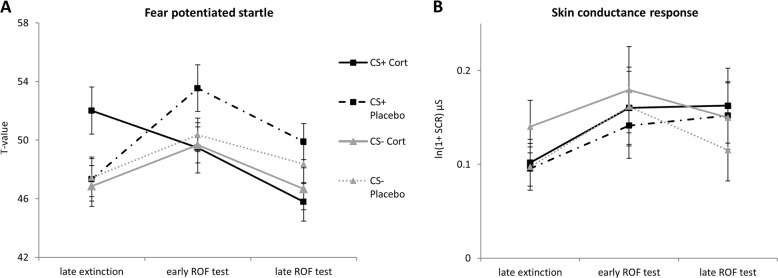
Physiological outcome measures. **a** Startle Response to CS+ and CS− at the end of extinction and during reinstatement test for cortisol and placebo groups **b** skin conductance response to CS+ and CS− at the end of extinction and during reinstatement test for cortisol and placebo groups

To examine if reinstatement led to a ROF in physiological measures and if this was moderated by cortisol administration, we conducted mixed design ANOVAs with the factors CS-Type (CS+, CS−), Time (late extinction (trials 4–6), early ROF-test (trials 1–3) and late ROF-test (trials 4–6)), and Group (cortisol, placebo). With regard to FPS, analyses revealed significant effects for CS-Type (*F*_1,39_ = 5.85, *p* = .020, *η*^2^ = .13), Time (*F*_2,78_ = 3.96, *p* = .023, *η*^2^ = .09), and for the relevant CS-Type*Time*Group interaction (*F*_2,78_ = 5.9, *p* = .004, *η*^2^ = .13) (see Fig. 4a). Post hoc analysis only showed significant ROF in the placebo group, as indicated by an increased FPS for the CS+ from late extinction to reinstatement (*p* = .026), whereas the cortisol group showed reduced FPS towards the CS+ from late extinction to late reinstatement (*p* = .017). Furthermore, the cortisol group did no longer show differential FPS for CS+vs. CS− (all *p*s < .15), whereas the placebo group did show a trend towards such differential FPS at the beginning of ROF test (*p* = .059)^6^. Explorative analysis with sex as additional factor did not reveal any sex-related cortisol effects with respect to FPS (all ps > .561). Regarding SCR, analysis did not find effects for CS-Type (*F*_1,40_ = 1.72, *p* = .197), Time (*F*_2,80_ = 1.88, *p* = .159), CS-Type*Time (*F*_2,80_ = 2.23, *p* = .115) and importantly no CS-Type*Time*Group interaction (*F*_*2*,80_ = 0.90, *p* = .410) (see Fig. 4b). Thus, our hypothesis was not confirmed with respect to SCR.

#### Cortisol levels during return of fear test

A mixed design ANOVA with the factors Time (arrival, pre, +0 min, +15 min, and +30 min) and Group (cortisol, placebo) yielded effects for Time (*F*_4,184_ = 6.31, *p* < .001, *η*^2^ = .12) and for Time*Group (*F*_4,184_ = 3.85, *p* = .038, *η*^2^ = .). Post hoc analysis showed elevated cortisol levels in the placebo group compared with the cortisol group (arrival: *p* = .012, pre: *p* = .004, +0 min: *p* = .006, +30 min: *p* = .014) with a natural decrease of cortisol concentration throughout the ROF test.

## Discussion

This study aimed to examine if cortisol administration facilitates the consolidation of extinction learning in a naturalistic fear-conditioning paradigm. The cortisol group showed less ROF as indicated by a lower US-expectancy for the CS+ and attenuated FPS for the CS+ in the ROF test as compared with the placebo group. Thus, our study is—to our knowledge—the first study in humans showing that cortisol facilitates the consolidation of extinction learning. This result integrates well with the findings that heightened cortisol levels enhance the success of exposure therapy in patients with anxiety disorders^18,19,21–23,39^ and PTSD^24,25^.

Importantly, our results extend previous research by shedding light on the mechanism by which cortisol enhances the success of exposure therapy. Cortisol has been hypothesized to enhance the success of exposure therapy by inhibiting the retrieval of fear memory and by enhancing extinction learning. Recently, two studies questioned the relevance of the retrieval inhibitory effect of cortisol in PTSD. They showed that cortisol administration does not inhibit the retrieval of intrusive memories^28,40^. Thus, the extinction enhancing effect of cortisol may have been responsible for the positive results in the exposure studies. The current data support the role of cortisol in the enhancement of extinction learning. Cortisol as an extinction enhancer offers the exciting opportunity to administer it subsequent to an exposure session, thereby avoiding the risk of consolidating an unsuccessful treatment session. However, it has to be noted that criteria for “successful” exposure sessions are still under debate. The influential emotional processing theory^41,42^ implies that significant reductions on behavioral, verbal, and physiological fear responses during exposure are indicators for success. However, it has been shown that physiologically within a session, fear reduction is not necessary for a successful treatment outcome^43^. In consequence, the inhibitory model of exposure therapy was developed^44^. It states that exposure is based on extinction learning that relies on secondary inhibitory learning about a new CS–US relationship. According to this model, exposure should violate threat expectations as profoundly as possible in order to decrease the associative strength between the original fearful CS–US association and increase the new association. Therefore, a successful exposure session should be one that results in low threat expectations. However, it remains open how the violation in threat expectation is best measured. Furthermore, the empirical evidence for the inhibitory model of exposure is not particularly strong and other findings directly contradict its conclusion. For example, recent clinical trials show that the reduction in reported fear during exposure is crucial for treatment success^45–47^. LeDoux and Hofmann^48^ have thus argued that subjective reports about fear decline are the best measure to assess exposure success. To summarize, there is a need of more research on the definition of markers for successful exposure sessions, especially the relationship between verbal reports, behavior, and physiological responses has to be further examined.

Interestingly, our results showed that cortisol influenced both US expectancy, an explicit measure of the appraisal of likelihood of subsequent threat, and FPS, an implicit measure of threat expectancy linked to amygdala influence on startle circuits. This is striking, because most previous studies investigating pharmacological treatment enhancers primarily showed an effect on explicit knowledge^36^. Thus, our data indicate that cortisol may be a quite pervasive enhancer of extinction learning, as it influences both explicit and implicit conditioning measures.

Indeed, recent research has shown that cortisol administered prior to extinction learning reduced activation in the amygdale-hippocampal complex and enhanced the related functional connectivity to the mPFC. All of these brain regions have been shown to be central to fear memory extinction and all of these regions express receptors to which cortisol binds. Thus, it is likely that cortisol influences extinction memory consolidation by a stronger inhibitory control of the mPFC^49^.

In this study, we developed a novel fear-conditioning paradigm using traumatic film clips as US. Previous studies have shown that traumatic film clips are powerful USs^26–29^. The strength of the present paradigm is that it has high ecological validity for PTSD and anxiety disorders, as well as high comparability to classic fear-conditioning studies. Thus, it offers a new research tool to investigate learning and memory processes underlying PTSD and other anxiety disorders.

Although we nicely showed fear acquisition, extinction and ROF, there were some inconsistencies in the results. First, there was no extinction learning for valence of the CS. This is in line with previous findings, showing that evaluative learning is quite resistant to extinction^50,51^. Second, we did not find a group difference regarding SCR during ROF test, which might be due to an interfering influence of the startle probe, i.e., we assume that the expectancy of the aversive startle probe led to a generally higher arousal level in our participants and thus made it impossible to detect differences between the two conditions. To control for this, future studies should assess SCR without FPS. A further improvement of the paradigm would be to employ other measures of the strength of extinction memory such as generalizability or renewal effects as they play an important role in the success of psychotherapy.

One limitation of our study is that our sample consisted of healthy participants without any psychopathology, limiting generalizability to PTSD and anxiety disorders. Furthermore, we decided to only include women taking oral contraceptives in our study, which can be viewed as a limitation. We decided to include oral contraceptives users for two reasons: (1) PTSD is more common in women than in men^25^ and even though the use of hormonal oral contraceptives is declining, it is still one of the most frequently used contraceptive methods. (2) Since this is the first study investigating the influence of cortisol on extinction memory, we tried to keep the experimental design as simple as possible. Nevertheless, future studies should extend these findings to free-cycling women, because it is known that neural effects of highly aversive films^33^, emotional memory formation in general^52^, and cortisol effects on memory^31^ differ between free-cycling women and women taking hormonal contraceptives. Furthermore, we did not control whether our female participants were in the active pill-intake phase or in the pause phase, which is followed by a slight change in hormone concentrations^53^.

In summary, the present study extends the knowledge about the enhancing effects of cortisol on exposure therapy by showing that cortisol facilitates the consolidation of extinction learning. Our results may have important implications for the employment of cortisol in the treatment of PTSD and anxiety disorders.

## Supplementary information

Supplementary Material.
