# Supplementary material for: Cortisol administration after extinction in a fear-conditioning paradigm with traumatic film clips prevents return of fear
Source: Transl Psychiatry. 2019 Apr 8;9:128. doi: 10.1038/s41398-019-0455-0 (PMC6453889; doi:10.1038/s41398-019-0455-0)
Supplement: Supplementary file 1 — Supplementary Material. [file 41398_2019_455_MOESM1_ESM.pdf]

Supplementary material

Detailed information about the physiological measures

Fear potentiated startle (FPS). Two flat electrodes were positioned below the left eye on the orbicularis oculi for the recording of the startle reaction. The audio files were reproduced with a dynamic audiometer headphone from Holmco (Modell PD81). Data was recorded with two channels at 22050 samples and 15 bits. The obtained raw data were smoothed with a 15.9Hz low-pass filter, a 25Hz high-pass filter and a 50Hz notch filter. Startle data were then manually inspected to mark the startle reactions and to edit the signal regarding measurement errors and artefacts. The amplitude values were calculated relative to the baseline of the electromyography (EMG) signal 50ms before the trigger onset in accordance to the guidelines for onset latency (Blumenthal et al., 2005). FPS responses were normalized by T-transformation. 4 participants were classified as non-responders with less than 70% valid trials and were excluded for further analysis regarding FPS.

Skin conductance response (SCR). Skin conductance was assessed using two Ag/AgCl electrodes filled with isotonic electrode gel. Electrodes were attached to the proximal part of the palm of the participants´ non-dominant hand (with an alternating current of 1mA synchronized with the sampling frequency passed between the electrodes). The raw signal was decimated to 25 Hz and then manually edited for artefacts and smoothed using a 1 Hz low-pass filter.

Film clips

**aversive film clips**	**neutral film clips**
127 Hour, ***Danny Boyle (2012)***	**Die Frau des Polizisten,** *Philip Gröning (2013)*
Antichrist, ***Lars von Trier (2009)***	**Drei Farben: Blau,** *Krzysztof Kieślowski (1993)*
Final Destination, ***James Wong (2000)***	**Drei Farben: Weiß,** *Krzysztof Kieślowski (1994)*
German Angst, ***Jörg Buttgereit, Andreas Marschall & Michal Kosakowski (2015)***	**Friendship!,** *Markus Goller (2010)*
Hostel, ***Eli Roth (2005)***	**Into the Wild,** *Sean Penn (2007)*
I spit on your grave 2, ***Steven R. Monroe (2013)***	**Wir Kinder vom Bahnhof Zoo,** *Ulrich Edel (1981)*
Scar, ***Jed Weintrob (2007)***	

Cortisol levels during ROF-test

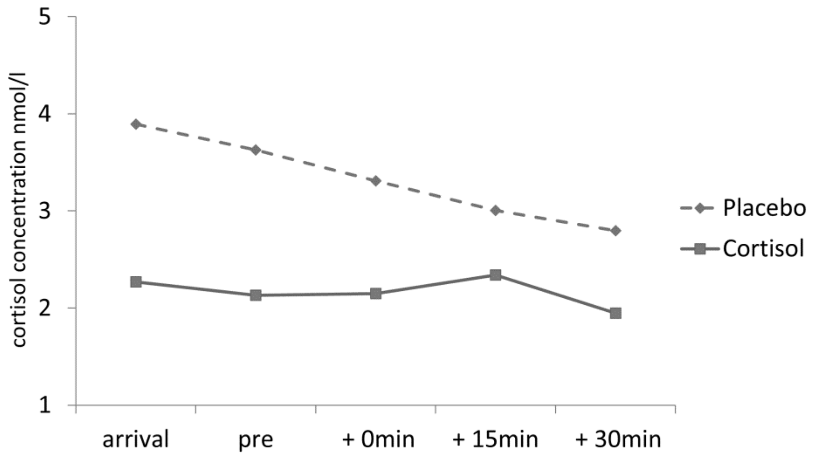

Fig. 5 Cortisol levels during ROF-test
